# Fluorescent emission profiles reveal interspecific differences in three Danube River Basin sturgeon species

**DOI:** 10.1038/s41598-026-45170-4

**Published:** 2026-03-25

**Authors:** Thomas Juhasz-Dora, Uros Ljubobratovic, Gyula Kovacs, Stein-Kato Lindberg

**Affiliations:** 1 Department of Aquaculture, Institute of Aquaculture and Environmental Safety, Hungarian University of Agriculture and Life Sciences, Páter K. U. 1., Gödöllő, 2100 Hungary; 2 Research Centre for Aquaculture and Fisheries, Institute of Aquaculture and Environmental Safety, Hungarian University of Agriculture and Life Sciences, Anna-Liget Str. 35, Szarvas, 5540 Hungary; 3 Centre of Clinical Documentation and Evaluation, Northern Norway Regional Health Authority, Tromsø, Norway

**Keywords:** Biological techniques, Ecology, Ecology, Ocean sciences, Zoology

## Abstract

Biofluorescence is widespread among marine teleosts but has not been reported in sturgeons (Acipenseridae). This exploratory study investigates interspecific differences in fluorescent emission profiles among three Danube River Basin sturgeon species using hyperspectral imaging. We examined 50 juvenile fish from 3 species: the Russian sturgeon (*Acipenser gueldenstaedtii;* *n* = 20), sterlet (*Acipenser ruthenus*; *n* = 20), and stellate sturgeon (*Acipenser stellatus;* *n* = 10). All species displayed green (~ 550 nm) and secondary red fluorescence (~ 615–730 nm) with a minor peak at ~ 650 nm under blue excitation light. Principal component analysis of standardized spectra revealed interspecific species-specific clustering, with PC1 (86.9% of variance) primarily reflecting differences in excitation light reflectance and differentiating stellate sturgeon, and PC2 (3.77% of variance) reflecting variations in green fluorescence intensity and distinguishing *A*. *gueldenstaedtii* from *A. ruthenus*. *A. ruthenus* exhibited higher fluorescence with lower excitation reflectance, while *A. stellatus* showed the reverse pattern, and *A*. *gueldenstaedtii* displayed consistently stronger emissions across shorter wavelengths (500–530 nm). These findings provide the first evidence of species-specific fluorescent spectral patterns in sturgeons. Such signatures may reflect differences in integument structure and pigment biochemistry, and they hold promise as non-invasive tools for species identification, aquaculture monitoring, and conservation management.

**Supplementary Information:**

The online version contains supplementary material available at 10.1038/s41598-026-45170-4.

## Introduction

Biofluorescence, the absorption of blue light and its re-emission at longer wavelengths, has been documented across a broad phylogenetic range of fishes, indicating that it is not restricted to a single lineage or ecological niche. Comparative surveys have shown that fluorescent emissions occur in distantly related taxa, a pattern consistent with repeated evolutionary emergence rather than inheritance from a common ancestor^1^. More recent phylogenetically explicit analyses with expanded taxonomic coverage further support this view, indicating that biofluorescence has arisen on the order of ~ 100 times independently across teleost fishes, with repeated gains and losses over evolutionary time^2,3^. Together, these studies indicate that biofluorescence represents an evolutionary labile trait in fish. While biofluorescence is prevalent in marine ecosystems, its documentation within freshwater temperate ecosystems remains rare. Notable exceptions highlighting the diversity of fluorescent mechanisms beyond classical Green Fluorescent Protein (GFP)-like proteins include the bilirubin-binding UnaG in Japanese eels (*Anguilla japonica*) and Sandercyanin in the North American walleye (*Sander vitreus*), a seasonal red-fluorescent protein (~ 675 nm) that forms as a biliverdin IXα-dependent tetramer^4,5^. Critically, sturgeons possess a relevant biochemical foundation for such mechanisms. Analysis of sturgeon bile has revealed a high proportion of bilirubin IXa conjugates, confirming active heme catabolism and the presence of potential fluorophore substrates^6^. Furthermore, recent research has successfully detected and monitored autofluorescence (e.g., from NADH and vitamin A) in sterlet (*Acipenser ruthenus*) fillets, linking spectral changes directly to spoilage and physiological status^7^. Recent studies have expanded this documentation of fluorescence to species produced in commercial aquaculture, such as the lumpfish (*Cyclopterus lumpus*), whose green fluorescent emissions responded to applied stressors typically experienced in captive conditions ^8,9^. Given these findings, the potential for similar fluorescence mechanisms in other phylogenetically distinct and ecologically specialized freshwater fish groups warrants investigation.

Sturgeons (Acipenseridae) represent an ancient lineage that has persisted through multiple mass extinctions. Today, the order Acipenseriformes (27 sturgeon species and 1 paddlefish species) are among the most threatened vertebrate groups globally, mainly due to anthropogenic interventions^10^. These fish exhibit distinctive physiological and behavioral adaptations for large lacustrine and riverine ecosystems^11^. Such complex habitats are required to fulfil life cycles^12^ that are complicated by migration, delayed maturation, and longevity^13^. The Danube River system historically hosted six sturgeon species ^11,14^. Preventing the disappearance of the remaining five species requires active protection and conservation measures. The ex-situ sturgeon live gene bank at the Hungarian University of Agriculture and Life Sciences, Institute of Aquaculture and Environmental Safety, Research Center for Fisheries and Aquaculture (MATE AKI HAKI) contains four of the five native Danube sturgeon species used for conservation and restocking efforts in the Middle and Lower Danube regions: Beluga (*Huso huso*), Russian sturgeon (*Acipenser gueldenstaedtii*), Stellate sturgeon (*A. stellatus*), and *A. ruthenus*.

The *A. gueldenstaedtii* is the most widely distributed large anadromous species in the Danube River basin^11^. Adults historically migrated up to 1200 km upstream from the Black Sea to spawn in distinct spring and autumn migrations^11,15^. The construction of the Iron Gate dams in Serbia cut off access to spawning grounds, collapsing all seasonal runs^11,14,16^. *A. gueldenstaedtii*, although mostly an anadromous species, also has smaller potamodromous populations in the Danube River system^11,14^. *A. stellatus* is considered a transitional species between potamodromous and anadromous ecotypes, with both juveniles and adults capable of moving across varying salinities^17^. Until the beginning of the twentieth century, *A. stellatus* was formerly widely distributed in the Black Sea and the Danube River floodplain^11,18^. *A. stellatus* has the third highest commercial value for caviar (Sevruga) after Beluga and Osetra (*A. gueldenstaedtii*) caviars^19^. As the smallest and only potamodromous species, the sterlet has a few self-sustaining populations remaining within the middle reaches of the Danube River^20^. However, dam construction destroyed suitable spawning and foraging habitats^21^, severely reducing population levels. Sturgeon species exhibit a high propensity for interspecific hybridization within the order Acipenseriformes. Numerous natural and artificial hybrids have been documented, both in wild populations and under aquaculture conditions^22^ ). *A. ruthenus* hybridize with both local and introduced sturgeon species^23^. These hybrids are often used in aquaculture because sterlets are small, mature early, and adapt well to captivity, making them a practical choice for selective breeding.

Despite their high commercial value, sturgeon aquaculture is constrained by several biological and management challenges. High-density rearing environments often induce chronic stress, impairing immune function and growth performance^24–27^. While hybridization can enhance production traits, it complicates stock identification and raises the risk of genetic introgression into wild populations^23,28^. Adding to these challenges, sturgeons exhibit cryptic stress responses that can be difficult to detect due to their thick, scaleless skin and bony scutes, limiting the timeliness of health interventions^29^. These same dermal structures may, however, interact with light in unique ways, potentially harboring previously undescribed photonic properties. Although biofluorescence has not yet been reported in sturgeons, their phylogenetic antiquity and ecological specializations make them intriguing candidates for such traits. The recent discovery of stress-responsive biofluorescence in *C. lumpus* suggests that similar pigment-mediated mechanisms possibly involving biliverdin or other chromophores^30–32^ could occur in sturgeons. If present, such fluorescence could serve as a non-invasive biomarker for real-time stress assessment and lineage verification, thereby addressing major bottlenecks in both conservation and farming. Hyperspectral imaging, already proven effective in detecting biofluorescent emissions in lumpfish at different life stages^8,9^, offers a promising tool for phenotypic differentiation and health monitoring in sturgeons without the need for physical handling. Sturgeon aquaculture and conservation are increasingly affected by interspecific hybridization, which complicates species identification based on external morphology alone. Although genetic methods provide definitive resolution, they are invasive and time-consuming, limiting their utility for rapid or repeated assessment of live animals. We therefore evaluated whether species-specific fluorescence emission patterns could serve as a rapid, non-invasive phenotypic marker to distinguish closely related sturgeon species.

This study investigates the occurrence of fluorescence in sturgeons (Acipenseridae) by examining emission profiles across three Eurasian sturgeon species: *A. gueldenstaedtii*, *A. ruthenus,* and *A. stellatus*, using hyperspectral imaging and multivariate analysis. Specifically, we aim to:

1. Determine whether fluorescent emissions occur within Acipenseridae, a freshwater genus of high economic and ecological significance
2. Characterize species specific fluorescent signatures in captive collections
3. Analyze interspecific variation in fluorescent emissions to assess their potential as taxonomic or physiological markers

## Results

### RGB photography

**Fig. 1:**
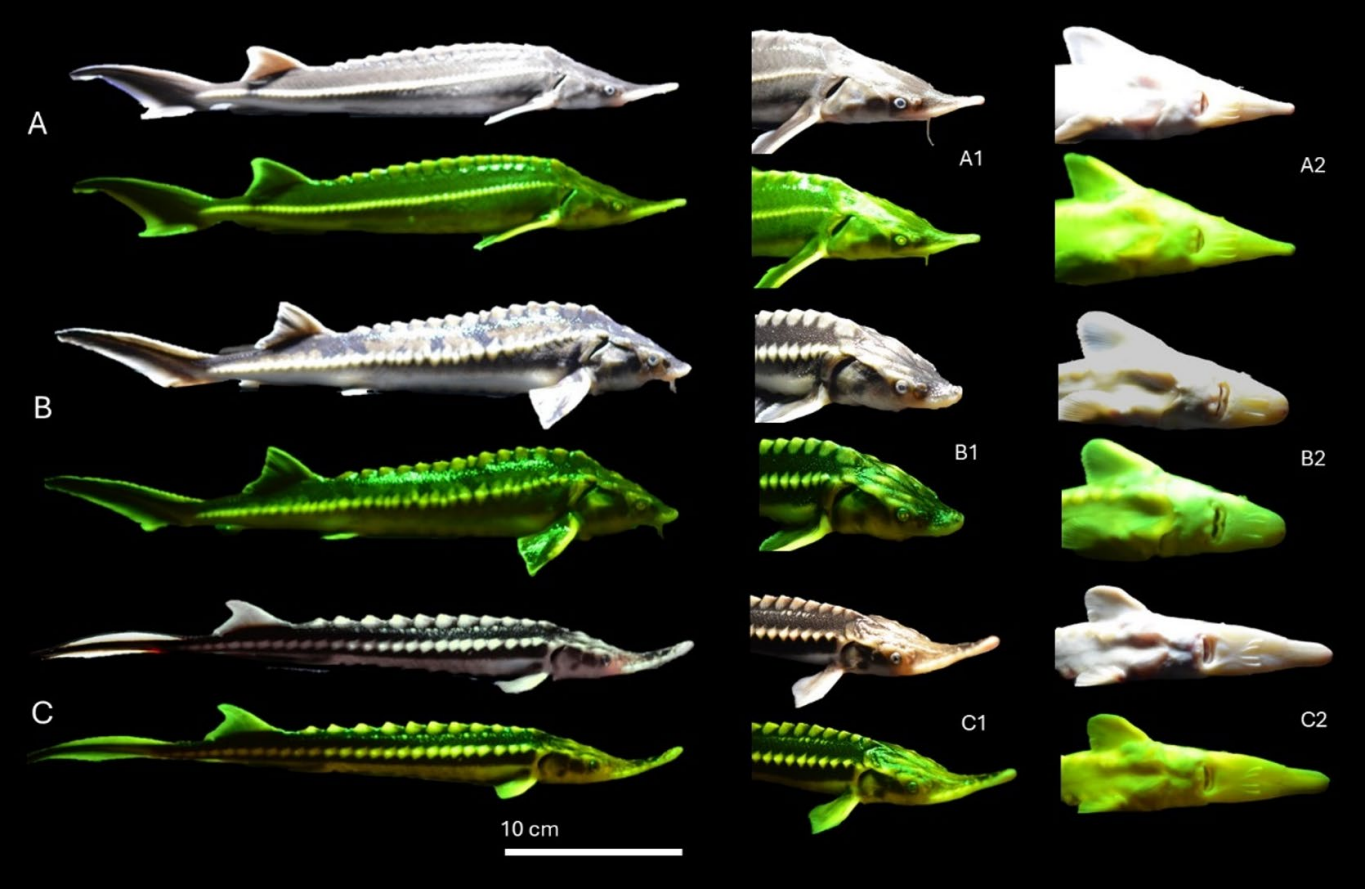
Interspecific variation in biofluorescence of three Eurasian sturgeon species. RGB photographs of sturgeons under cool white light and royal blue excitation light (~ 445 nm) reveal species-specific fluorescent emission patterns for (**A**, **A1**, **A2**) Sterlet (Acipenser ruthenus), (**B**, **B1**, **B2**) Russian sturgeon (Acipenser gueldenstaedtii), and (**C**, **C1**, **C2**) Stellate sturgeon (Acipenser stellatus). For each species, the main panel (**A**, **B**, **C**) shows the full body under excitation light. Insets (**A1**–**C1**) show detail of the rostrum and pectoral fin. Insets (**A2**–**C2**) show detail of the ventral cranio-abdominal region. Fluorescence is observed in all species, with visible emissions primarily localized to the rostrum, pectoral fins, abdomen, and along the dorsal and lateral scutes.

Biofluorescence produced by three sturgeon species was successfully captured using RGB photography under royal blue excitation lighting (~445 nm) and compared to their visible coloration under normal cool white light (Fig. 1). Distinct species-specific fluorescent emission patterns were observed. The sterlet exhibited a diffuse green fluorescence across the body, with particularly pronounced emissions along the rostrum, pectoral fin, abdomen, and the dorsal and lateral scutes. In contrast, the Russian sturgeon displayed a lower intensity green fluorescence, primarily localized to the denticle-containing regions of the dermis along the dorsal region. This dermis may contain concentrated levels of chromatophores that reduce fluorescent emissions. High emissions can be observed from the dorsal and lateral scutes, dorsal fin, ventral snout, and abdomen. The Stellate sturgeon also displayed strong green fluorescence along the dorsal and lateral lines, dorsal fin, caudal fin, rostrum, and pectoral fin. Notably, the ventral rostrum and abdomen of the Stellate sturgeon exhibited a unique fluorescent signature in the visible part of the spectrum that was distinct from the other two species.

### Raw spectra

**Fig. 2:**
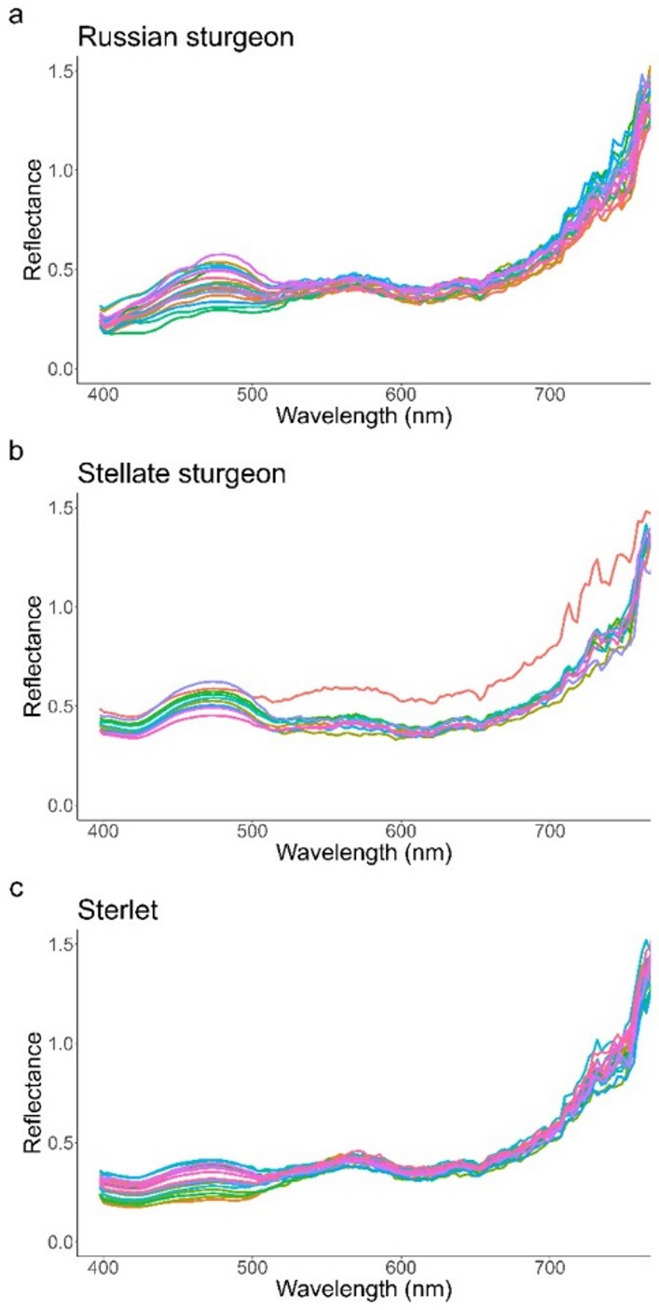
Reflectance spectra for Russian sturgeon (*Acipenser gueldenstaedtii;* *n* = 20) (**A**), Stellate sturgeon (*Acipenser stellatus;* *n* = 10) (**B**), and sterlet (*Acipenser ruthenus;* *n* = 20) (**C**) scanned under blue excitation lighting (~ 445 nm). A reflectance maximum in the green spectral region (~ 550 nm) is observed consistently across all specimens, followed by lower-amplitude spectral features and a broad increase in reflectance in the red part of the spectra (~ 650 nm; ~ 675–730 nm).

**Fig. 3:**
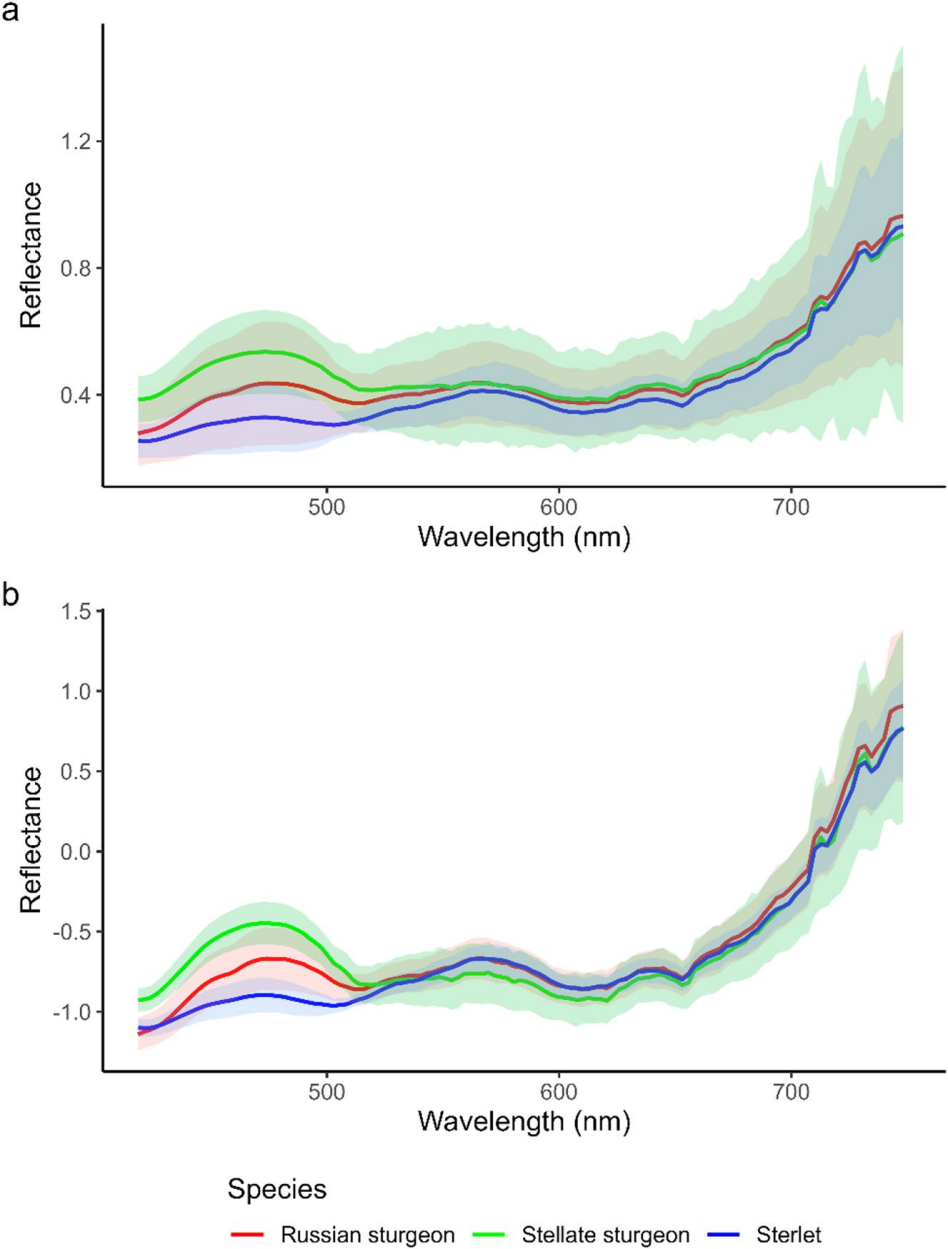
Reflectance spectra (**A**) and mean standard normal variate (SNV) transformed reflectance spectra (**B**) for each sturgeon species. The Stellate sturgeon (*Acipenser stellatus;* green) had the highest excitation peak (~ 450–500 nm) and the lowest fluorescent peak in the green (~ 550–600 nm) and red (~ 620–650 nm). Conversely, the sterlet (*Acipenser ruthenus*; blue) had the lowest excitation peak with green fluorescence peak matching that of *A. stellatus*. The Russian sturgeon (*Acipenser gueldenstaedtii*; red) had an excitation peak with a height between the other two species while producing a fluorescent signature like that of *A. ruthenus*. Shaded regions represent ± 1 standard deviation around the mean spectrum for each species.

**Fig. 4:**
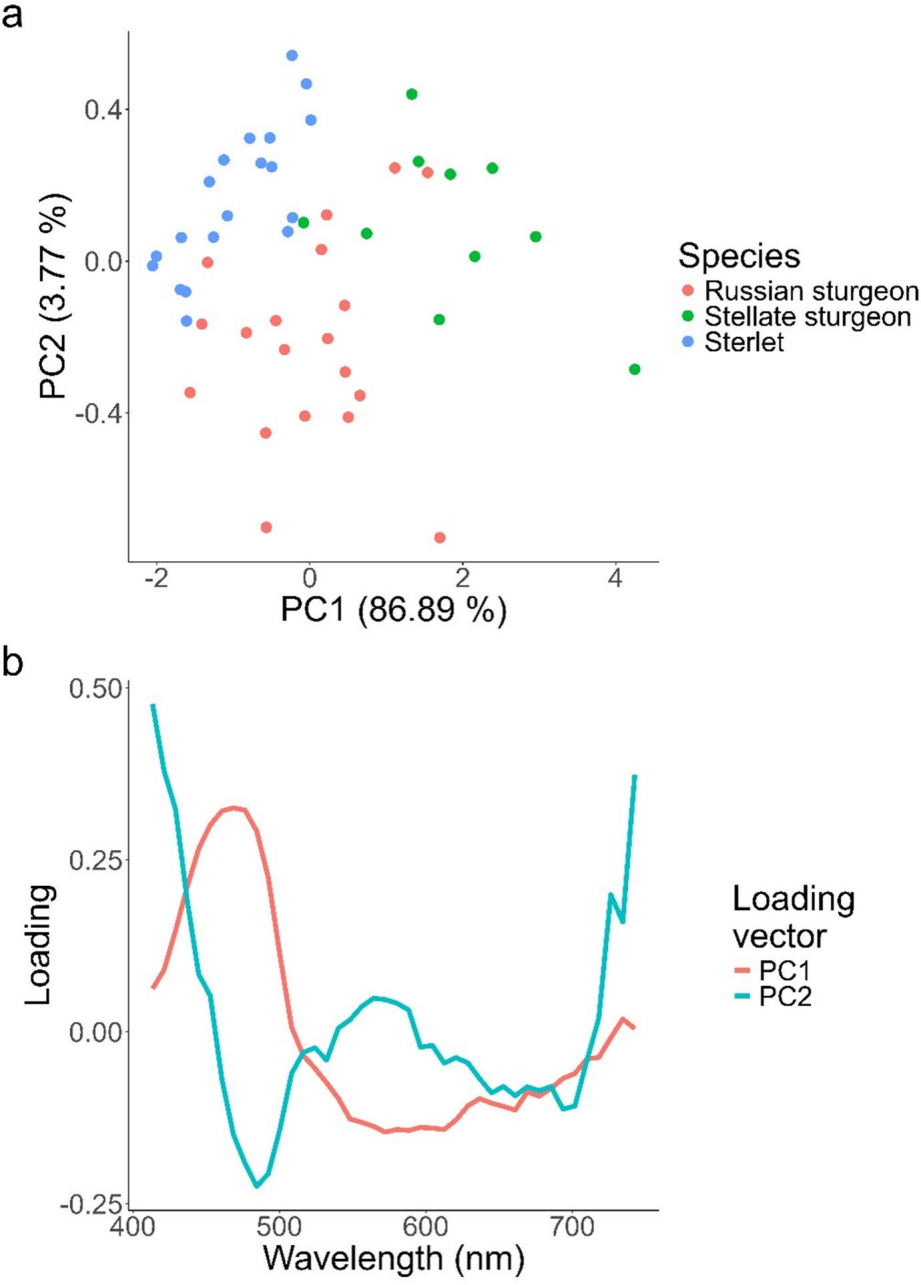
Scatter plot (**A**) and loading plot (**B**) of the first and second principal component of the standard normal variate (SNV) transformed mean reflectance spectra per Eurasian sturgeon species. The first principal component (PC1) accounts for most of the variation and can be interpreted as the reflected excitation light judging by its loading. The second principal component (PC2) is influenced primarily by wavelength bands within the green to red spectral regions and is consistent with variation associated with fluorescence emission rather than excitation reflectance.

The raw untransformed mean reflectance spectra per individual exhibited consistent characteristics across species groups (Figs. 2, 3 and 4). A primary peak (~400 to 500 nm) corresponded to excitation light emissions, where the smoothness of the curve reflects the high signal-to-noise ratio (SNR) present. A secondary peak (~500-600 nm) indicates a green fluorescence emission. The line contains more noise since the fluorescence emission was weaker by orders of magnitude, and the SNR correspondingly lower. A third, smaller peak appeared between 615 and 650 nm, representing a lower secondary fluorescence peak in the red spectra. The observed dip at the peak’s right edge likely represents an artifact of the white reference’s spectral properties rather than a true fluorescence feature, as fluorescence peaks typically exhibit broad, symmetrical distributions that would extend further before reaching a trough. Fluorescence peaks are usually broad and symmetrical, and the right upper edge of this red peak should therefore extend further before hitting a trough. The secondary broad slope in the red part of the spectra continues until ~750 nm, where the reflectance values exceed 1.0. This shows that sturgeons are more reflective in this spectral region than the white reference standard. In the far-red to near-infrared range (>750 nm), the signal intensity falls below the camera’s noise floor. This is expected given both the minimal illumination in this spectral region and the white reference’s inherently low reflectance at these wavelengths. The combination of these factors results in signal degradation where meaningful spectral information can no longer be resolved.

### Principal component analysis

The spectral analysis reveals distinct interspecific differences in reflectance and fluorescence patterns among sturgeon species. *A. stellatus* demonstrates significantly higher reflectance of excitation light (400–500 nm) coupled with proportionally reduced green fluorescence emission (500–600 nm; Fig. 4). In contrast, *A. ruthenus* exhibits the inverse relationship, showing lower excitation light reflectance but enhanced fluorescence intensity. *A. gueldenstaedtii* presents an intermediate pattern between these two species. These observations align with fundamental energy conservation principles, where increased reflectance of excitation wavelengths necessarily corresponds to decreased fluorescence emission, as the energy involved in these two processes comes from the excitation light and must be conserved. The negative correlation between excitation light reflectance and fluorescence emission intensity observed across all three species supports this physical relationship.

The scatter plot of the first two principal components (PC1–PC2) reveals distinct clustering patterns among the three sturgeon species (Fig. 4). *A. stellatus* separates clearly from *A. gueldenstaedtii* and *A. ruthenus* along PC1, while the latter two species differentiate primarily along PC2. Examination of the corresponding loading plots indicates that PC1 is dominated by positive contributions from the excitation wavelength range (~400–500 nm), consistent with variation in excitation light reflectance. In contrast, PC2 is influenced primarily by wavelength bands within the green (~550–600 nm) and red (~650–730 nm) spectral regions associated with fluorescence emission. The excitation band contributes positively to PC1 and negatively to PC2, while a secondary loading maximum on PC2 corresponds to fluorescence-associated wavelengths. Together, these patterns indicate that PC1 captures variation related to excitation light interaction, whereas PC2 reflects interspecific differences in fluorescence emission structure.

### RGB image analysis

**Fig. 5:**
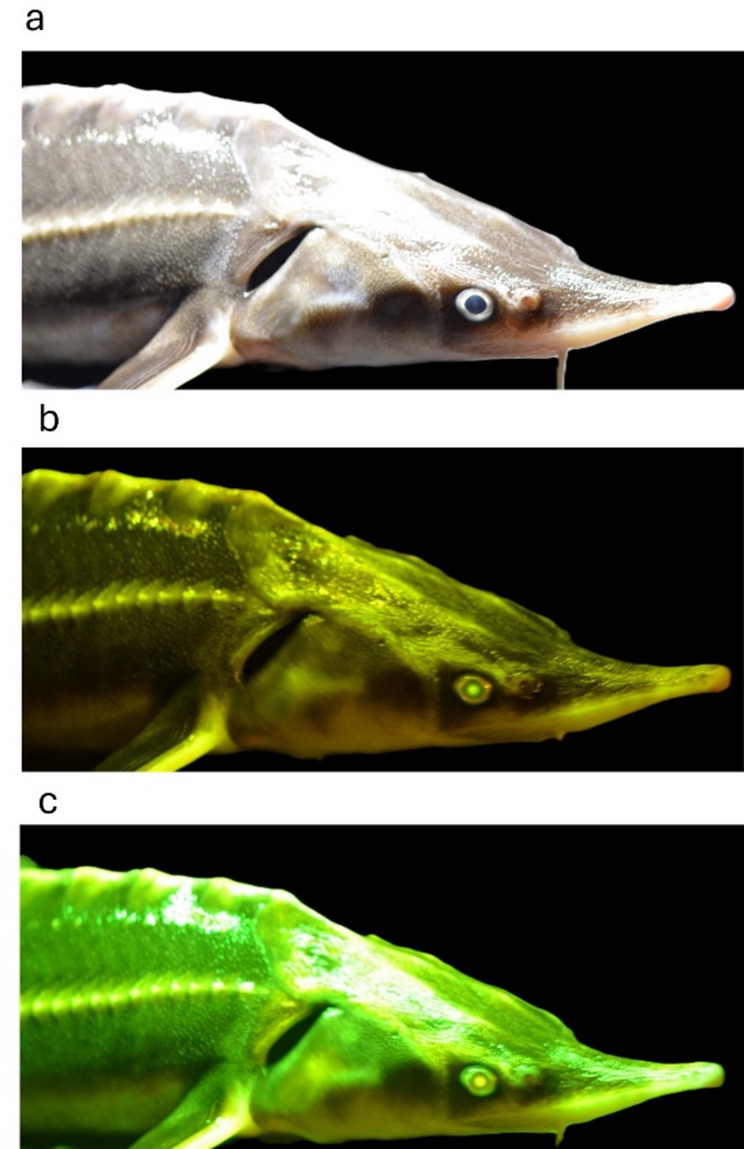
RGB images of a juvenile sterlet (*Acipenser ruthenus*) using cool white light (**a**), UV light (**b**) and royal blue light (**c**). The background has been set to pixel value zero to isolate fish pixels for histogram analysis. A yellow filter was used when taking photos under UV (~ 395 nm) and royal blue light(~ 445 nm).

**Fig. 6:**
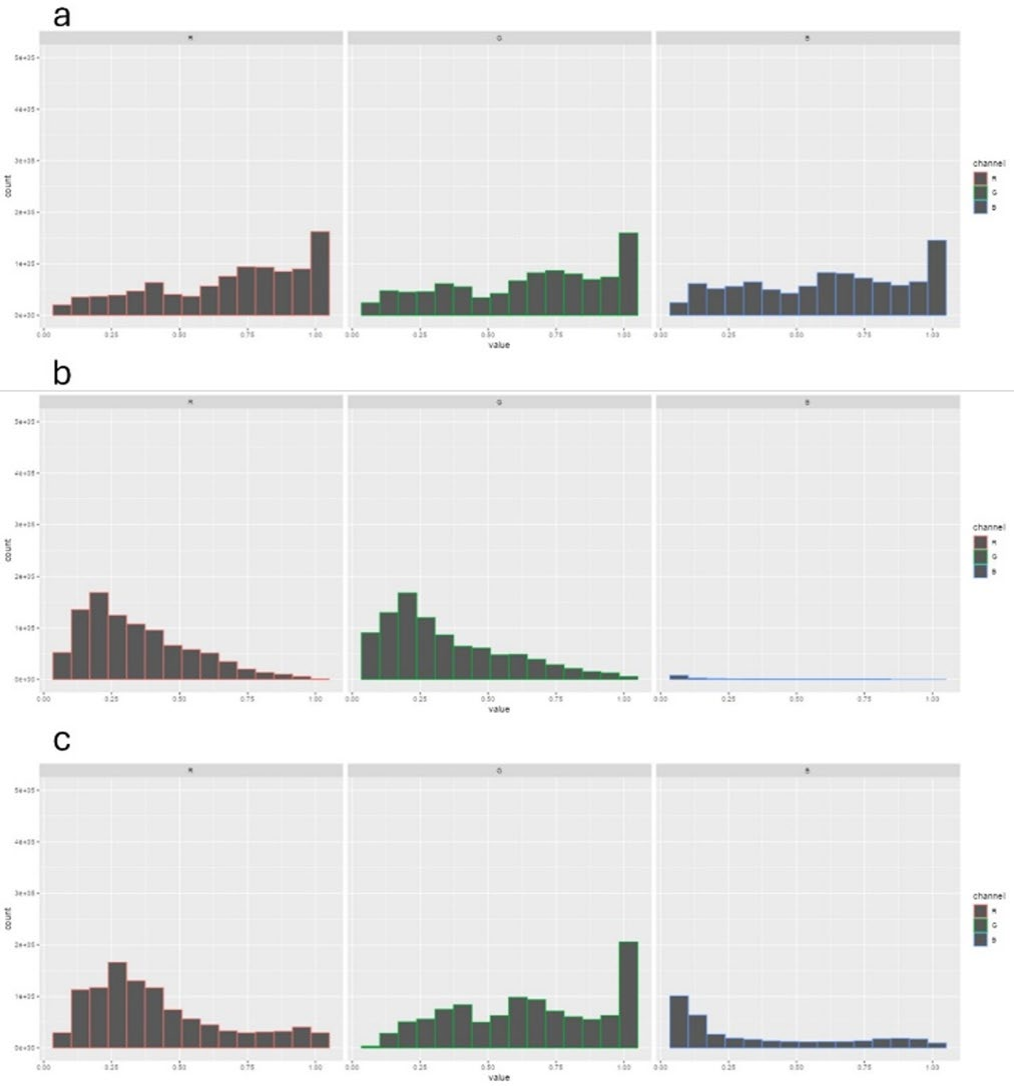
Histograms of pixel-intensity distributions for sterlet (*Acipenser ruthenus*) images to panels (**a**), (**b**), and (**c**) in Fig. 5 shown as an illustrative example of color-channel responses under different illumination conditions. The cool white light results in mostly flat histograms in all the color channels. Under UV light with a yellow filter the pixels are almost exclusively red and green with a peak towards the lower end of the pixel value range. Under royal blue light with a yellow filter there are some pixels with low values in the blue channel and a red pixel distribution with a peak around 0.25 as well as some higher values in the range 0.9 to 1.0 that were not observed under UV light. In the green channel the rightmost bar is the highest.

*A. ruthenus* imaged under the three illumination conditions is shown as a representative example. The photos are presented in Fig. 5, and the corresponding histograms of the pixel values for the three-color channels are shown in Fig. 6.

Under cool white light the histograms are similar for all three channels and are mostly flat. Under UV light there is equal parts red and green pixel values with a peak around 0.2 and a broad distribution skewed to the left. The royal blue produced a different response with a red channel histogram similar to the UV light, except there were some pixels in the far upper range. The green channel histogram has a bimodal shape with the tallest bar being the rightmost around pixel value 1.0. There were some pixels with blue values towards the lower parts of the range, which presumably were just past the cutoff wavelength of the yellow filter.

Although the fish being photographed is highly reflective, assuming that the UV and royal blue illumination does not have major components of red and green light, there should not be a high number of pixels with green and red values in the images taken under such illumination. This light could thus only originate from the sample itself, which indicates that the phenomenon being observed is indeed fluorescence. This could be observed for all three species.

## Discussion

This study provides the first evidence that biofluorescence occurs within Acipenseridae, a freshwater lineage of high ecological and economic importance. Using hyperspectral imaging under controlled excitation conditions, we identified reproducible interspecific differences in fluorescence emission profiles among juvenile Eurasian sturgeons reared in aquaculture. All three species exhibited measurable green and red fluorescence components, with species-specific differences in spectral shape rather than reliance on single wavelength features. Unsupervised principal component analysis confirmed clear species-level clustering, demonstrating that biofluorescence represents a reproducible, species-dependent phenotypic trait in these taxa. In this exploratory framework, interspecific differences were evaluated statistically at the level of whole spectral profiles using multivariate analysis, rather than through post hoc testing of individual excitation or emission peaks, which would require predefined hypotheses and independent validation. Fluorescence was spatially heterogeneous and consistently concentrated in anatomically distinct regions, including scute ridges, the rostrum, and ventral areas, indicating that fluorescence expression is structured rather than diffuse across the integument.

The observed spatial heterogeneity in fluorescence can be interpreted in the context of sturgeon integumentary anatomy. Unlike most teleost fishes, sturgeons lack elasmoid scales and instead possess a thick, armor-like dermal layer reinforced with mineralized bony scutes, denticles, and dense collagen matrices^33^. These scutes differ markedly from surrounding skin in their composition, thickness, and organization, resulting in pronounced structural heterogeneity across the body surface. From an optical perspective, such heterogeneity is expected to modulate light propagation within the integument, as increased scattering and refraction within layered and mineralized tissues can prolong photon paths near superficial chromophores and structural reflectors, potentially enhancing localized fluorescence signals. In addition, the lateral line system in sturgeons is closely associated with modified scutes and canal-bearing dermal structures^34^, introducing further spatial variation in tissue architecture that may influence optical signal propagation. Regional variation in epidermal mucus thickness and composition, known to affect optical measurements in fish, likely modulates excitation light penetration and the escape of emitted fluorescence across the sturgeon’s complex dermal landscape^35,36^. Together, these anatomical features provide a plausible structural context for the observed concentration of fluorescence in specific body regions, without implying a specific molecular mechanism.

Beyond structural effects, the spatial clustering of fluorescence along scute ridges and rostral margins suggests additional hypotheses related to chromatophore composition and pigment biochemistry^35^. In particular, iridophores may contribute to localized fluorescence patterns. Guanine-based crystalline platelets within these cells are highly reflective^37^, and their orientation can modify local light fields, potentially creating conditions favorable for excitation light capture and emission visibility. However, the presence, density, and orientation of iridophores in specific sturgeon integumentary regions were not directly assessed in this study, and their role in fluorescence expression therefore remains a hypothesis requiring targeted histological and biochemical validation.

Similarly, bile pigment–related pathways represent a plausible biochemical link between fluorescence and physiological state. Biliverdin and related tetrapyrroles are established fluorophores in several fish systems, most notably within the Anguilliformes, where bilirubin-binding proteins drive bright green fluorescence^38,39^. Recent analytical protocols have also validated the use of high-sensitivity fluorometric assays for the combined determination of these pigments in biological matrices^40^. Moreover, stress-related modulation of biliverdin-associated fluorescence has been demonstrated previously^41^. While such pathways are consistent with the spectral characteristics observed here, the present study was not designed to identify specific fluorophores or resolve underlying metabolic mechanisms. Accordingly, fluorescence should not be interpreted as a stress biomarker in sturgeons without explicit validation linking fluorescence variation to established physiological stress indicators (e.g., oxidative stress markers or endocrine responses).

The species-specific fluorescence patterns identified in this study suggest potential applications in sturgeon aquaculture and conservation, particularly for non-invasive phenotyping. One prospective application is species identification in hatchery settings, including early-life-stage discrimination. Given the high prevalence of natural and artificial hybridization among sturgeon species, driven in part by polyploidy and weak reproductive isolation^42,43^, fluorescence-based phenotyping could complement existing morphological and genetic approaches where rapid, non-lethal screening is advantageous. However, the ability of fluorescence signatures to reliably distinguish hybrids from parental species remains untested and would require validation using known hybrid crosses and expanded sample sizes. In addition to species-level differentiation, preliminary observations indicate that fluorescence patterns may also vary with sex in sturgeons. External sex identification in juvenile and subadult sturgeons is notoriously difficult due to limited sexual dimorphism, and current methods rely on invasive or semi-invasive techniques such as ultrasound, biopsy, or endocrine profiling. During exploratory imaging, consistent differences in the spatial distribution and intensity of fluorescence were observed in regions associated with cranial and ventral anatomy in individuals of known sex; however, these observations were not quantified systematically and fall outside the formal scope of the present study. Targeted investigations combining fluorescence imaging with independent sex validation will be required to determine whether fluorescence-based phenotyping could contribute to non-invasive sex identification in sturgeons. Fluorescence-based physiological monitoring represents a second potential application. For such use, robust correlations between fluorescence changes and established physiological indicators, such as oxidative stress markers, bile pigment concentrations, or endocrine responses, would need to be demonstrated. Without such validation, fluorescence should not be interpreted as a direct proxy for stress but rather as a candidate phenotypic signal warranting further investigation.

Several limitations of the present study should be acknowledged. First, all individuals examined were juveniles, and it remains unknown whether fluorescence characteristics are conserved across ontogenetic stages or change with growth, maturation, and age-related modifications of integumentary structure and pigment composition. Developmental shifts in body size, scute morphology, chromatophore distribution, and metabolic state may all influence fluorescence expression. Future studies incorporating subadult and adult sturgeons will therefore be required to assess the stability of species-specific fluorescence patterns across development. Second, the smaller sample size for *A. stellatus* reflects practical constraints imposed by body size and live-animal imaging geometry and may influence the stability and generalizability of clustering results for this species. Expanding sample sizes and imaging capacity for larger-bodied sturgeons will be important in future work to further validate interspecific fluorescence differentiation.

These future efforts are particularly relevant for sturgeon conservation and aquaculture, where non-invasive phenotyping tools are urgently needed to support genetic integrity, welfare monitoring, and informed broodstock management. If validated across life stages and genetic backgrounds, fluorescence-based phenotyping could complement existing morphological and genetic approaches for managing hybridization risk in captive breeding and restoration programs.

By establishing that biofluorescence occurs within Acipenseridae and characterizing species-specific spectral profiles in juvenile Eurasian sturgeons, this study demonstrates that fluorescence encodes biologically structured variation in an ancient freshwater fish lineage. The observed interspecific differences and spatial heterogeneity indicate that fluorescence is not a trivial optical artifact, but a reproducible phenotypic property linked to integumentary structure and light–tissue interactions. While the mechanistic basis and developmental stability of these signals remain to be resolved, the present findings position hyperspectral fluorescence imaging as a viable, non-invasive approach for investigating phenotypic diversity, species differentiation, and physiological state in sturgeons under controlled and applied conditions.

## Methodology

### Experimental animals and husbandry

This study examined 50 juvenile sturgeons from the 2024-year class, compromising three species: 20 *A. gueldenstaedtii*, 20 *A. ruthenus*, and 10 *A. stellatus*. *A. stellatus* is highly susceptible to handling stress; therefore, the smaller sample size for this species (*n* = 10) reflects physical constraints imposed by animal size and welfare considerations. Larger individuals exceeded the effective field of view and handling geometry of the hyperspectral imaging system and could not be imaged reliably under live-animal conditions without compromising welfare for this stress-sensitive species. All fish originated from a live gene bank established at MATE AKI HAKI in Szarvas, Hungary, established to support research and conserving the genetic resources of sturgeon species occurring in the Danube Basin. Sturgeons were reared in indoor Recirculating Aquaculture System (RAS) 2 m^3^ tanks under controlled conditions (temperature 18–21 °C, dissolved oxygen > 6 mg/L, pH 7.0–7.5). Systems were equipped with mechanical (drum filters) and biological filtration, ozone sterilization, and temperature control. Juveniles were fed formulated feeds (Aller Bronze, Aller Aqua Group, Denmark) according to size and nutritional requirements. Effective field-of-view and handling geometry of the hyperspectral imaging system and could not be imaged reliably within live-animal welfare constraints.

Juvenile groups had the number of individuals and total biomass recorded. *A. ruthenus* had a mean body weight of 189 g and a mean total length of approximately 38 cm. *A. gueldenstaedtii* had a mean body weight of 231 g and a mean total length of approximately 36 cm. *A. stellatus* had a mean body weight of 412 g and a mean total length of approximately 52 cm.

Fish were fasted for 24 h prior to imaging, following standard aquaculture and experimental handling practice, to reduce metabolic variability and limit potential dietary influences on fluorescence signals. Prior to scanning, sturgeons were anesthetized using 2-phenoxyethanol (ORPC, Italy) at a concentration of 0.3 mL L^−1^, consistent with standard protocols for the handling and short-term immobilization of juvenile sturgeons, to minimize handling stress and ensure animal welfare. All handling and imaging procedures were conducted with a strict out-of-water time limit of under 60 s per individual. Fish were randomly selected by a technician and scanned individually on 15 July 2025.

All procedures involving live animals were approved by the Institutional Animal Welfare Committee of the Hungarian University of Agricultural and Life Sciences Szent István Campus (permission number: MATE-SZIC/1718–1/2022, date of approval: 31 July 2022) and were conducted in accordance with relevant national and institutional animal welfare regulations.

### Instrumentation

Imaging was performed using a line-scanning hyperspectral camera (FX10, Specim Spectral Imaging Ltd., Oulu, Finland) operating in the visible and near-infrared (VNIR) range (400–1000 nm). The spectral resolution was 5.5 nm and 1024 spatial pixels were across the scan line. The camera was mounted at a working distance of 50 cm, yielding a spatial resolution of 0.2 mm/pixel. To optimize the signal-to-noise ratio for the target fluorescence emissions, analysis was focused on the 418–750 nm range. Spectral binning (2 ×) was applied, reducing the spectral resolution to ~ 11 nm to increase signal strength; spatial binning was maintained at 1 × to preserve fine-scale features. A custom motorized translation stage moved samples at a constant speed of 0.2 m/s. Image acquisition was synchronized to this motion at a line rate of 50 Hz with an exposure time of 5 ms per line.

Illumination was provided by a high-intensity LED array (G5 XR30 Pro Radion, Ecotech Marine, Bethlehem, PA, USA) configured to emit royal blue light centered at ~ 445 nm for fluorescence excitation. The blue LED excitation source exhibited a dominant emission peak in the blue spectral range. The excitation spectrum was dominated by this blue emission peak, and no band-pass filter was applied at the source. Fluorescence analysis therefore focused on wavelength-shifted emission outside the excitation peak, with background correction and relative intensity-based comparisons used to minimise contributions from reflected excitation light. The royal blue LED channel used for excitation does not emit in the infrared range, and no dedicated infrared LEDs were present in the illumination system; furthermore, signal intensity beyond ~ 750 nm fell below the camera noise floor, confirming that the observed red-range features are not attributable to infrared reflectance. This absence of measurable infrared contribution is illustrated in Supplementary Figure S1. All fluorescence imaging was acquired in a darkened room to minimize ambient light contamination.

RGB (Red Green Blue) fluorescence images were acquired following the same optical documentation methodology used in previous biofluorescence studies^41,44–46^. Representatives of each species were photographed under ambient white illumination and under short-wavelength excitation using both ultraviolet light (~ 395 nm) and royal blue light (~ 445 nm) generated by a G5 XR30 Pro Radion LED lighting system (Ecotech Marine, Bethlehem, PA, USA).

RGB images presented for sturgeon were captured using a digital single-lens reflex (DSLR) camera (Nikon D5100) equipped with an AF-S Micro-Nikkor 60 mm f/2.8G IF-ED lens (Nikon Corporation, Minato City, Tokyo, Japan). For all excitation-light images, a long-pass yellow barrier filter (Tiffen Yellow 12/62DY15; Tiffen Company, Hauppauge, NY, USA) was mounted in front of the camera lens to block reflected excitation light and allow only wavelength-shifted emission to be recorded.

### Hyperspectral analysis

Hyperspectral image processing and analysis were performed using the Breeze software (Prediktera, Umeå, Sweden). Prior to analysis, all images were calibrated to relative reflectance. A dark reference image (acquired with the lens capped) was subtracted to remove dark current and electronic offset. Each image was then divided by a white reference image acquired from a polytetrafluoroethylene (PTFE) calibration panel (Labsphere, Inc., North Sutton, NH, USA) to correct for non-uniformity in the illumination source and sensor sensitivity.

**Fig. 7:**
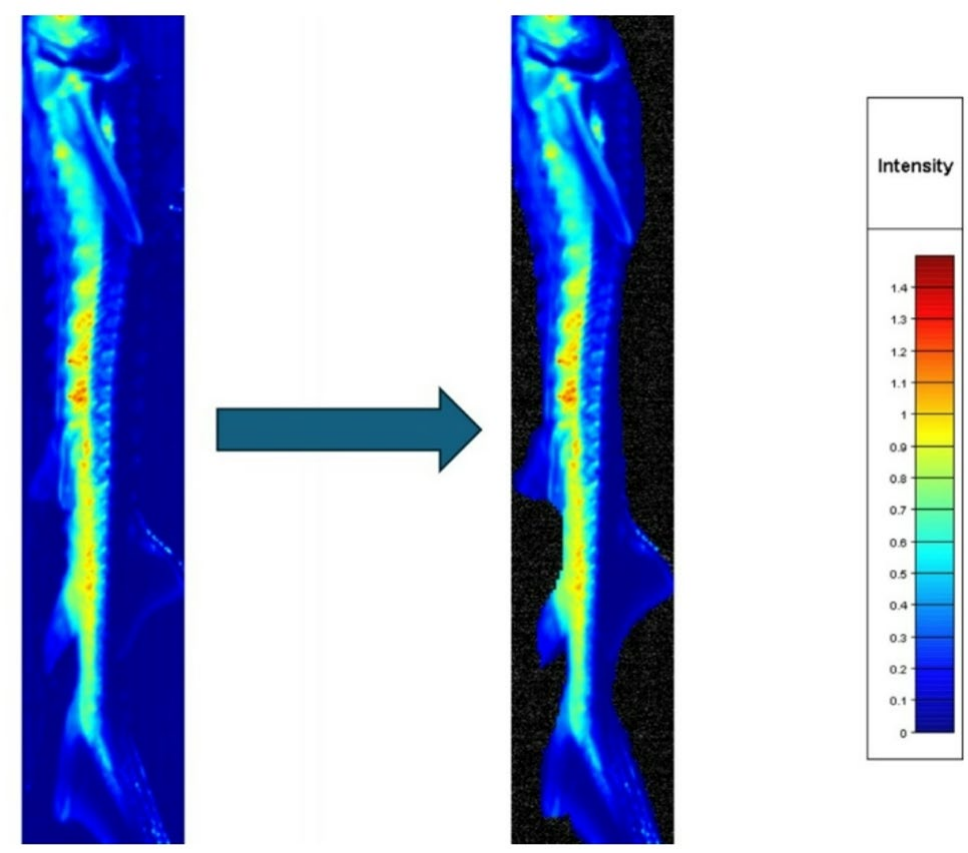
Example of raw and segmented hyperspectral image of a juvenile Russian sturgeon (*Acipenser gueldenstaedtii*), illustrating background masking applied prior to pixel-based analysis. Pixel values represent reflectance intensity at 445 nm.

It is important to note that while this calibration is standard for reflectance measurements, its application to quantitative fluorescence intensity is limited. The white reference standard corrects for the intensity of the excitation light (445 nm) but not for the variable quantum yield of fluorescence emission. Therefore, the reported values represent apparent reflectance, and subsequent analyses focus on the shape and relative differences of the spectral profiles rather than absolute fluorescence intensities (Fig. 7). Accordingly, fluorescence in this study refers to wavelength-shifted emission detected outside the primary excitation peak and analysed comparatively across individuals and species, rather than absolute fluorophore-specific emission intensities or quantum yields.

For each calibrated image, the fish was manually segmented from the background. Segmentation was used solely as a preprocessing step to isolate fish pixels from the background and was not treated as an analytical output. All segmentations were performed using a consistent workflow, and each segmented image was visually inspected to confirm anatomical completeness and absence of background artifacts prior to spectral averaging. Because segmentation outputs were not compared quantitatively between individuals or species, and downstream analyses were based on mean spectra aggregated across thousands of pixels per individual, minor boundary differences are unlikely to influence the observed interspecific patterns. We therefore did not apply formal inter-observer reproducibility metrics, which would be required for segmentation-focused studies but were beyond the scope of this exploratory analysis.

The mean spectral signature for each individual sturgeon was calculated by averaging the reflectance values of all pixels within the segmented region across all spectral bands. The analysis was constrained to the 418–756 nm range, where the signal-to-noise ratio was sufficient for robust analysis. To minimize artefacts associated with low signal-to-noise regions, spectral bands beyond ~ 750 nm, where signal intensity fell below the camera noise floor, were excluded prior to preprocessing. The resulting mean spectra were preprocessed using Standard Normal Variate (SNV) transformation to minimize light-scattering effects caused by surface topography and scale structure. This transformation centers each spectrum by subtracting its mean and scales it by dividing by its standard deviation. Because excitation reflectance, photon absorption, and fluorescence emission are physically coupled processes, SNV preprocessing does not decouple excitation and emission mechanisms, nor does it correct for tissue optical properties such as penetration depth or inner filter effects. Accordingly, SNV was applied strictly as a variance-normalization step to facilitate comparison of relative spectral shape across individuals and species, rather than to infer excitation efficiency, fluorescence yield, or excitation–emission energy transfer.

To investigate interspecific spectral differences, a Principal Component Analysis (PCA) was performed on the SNV-transformed spectral data matrix. The first two principal components (PCs) were retained for further interpretation. The score plot was examined to visualize clustering of individuals by species, and the corresponding loading plots were analyzed to identify the spectral wavelengths contributing most to the observed variance. PCA was applied as an unsupervised, variance-based dimensionality reduction method and therefore does not require assumptions of normality or homoscedasticity. Principal component retention was guided by inspection of scree plots and the proportion of variance explained, with PC1 and PC2 jointly capturing the majority of spectral variance and providing clear interspecific separation. Subsequent interpretation focused on loading patterns to identify wavelength regions contributing most strongly to interspecific differences.

### RGB image analysis

The RGB images were segmented using the Microsoft Photos app to set all background pixels to zero. The resulting images were downsized by 50 percent, and histograms for the red, green and blue channels were calculated with a pixel value threshold of 0.05 and 15 bins in the pixel value range 0 to 1.

## Supplementary Information

Below is the link to the electronic supplementary material.

Supplementary Material 1

## Data Availability

Raw hyperspectral image data are stored in native ENVI-compatible formats generated by the Specim FX10 system. Processed spectral data and principal component analysis (PCA) outputs are available in comma-separated values (.csv) format. All datasets generated and analyzed during this study are available from the corresponding author upon reasonable request and are provided for non-commercial research purposes.
