# Supplementary material for: Fluorescent emission profiles reveal interspecific differences in three Danube River Basin sturgeon species
Source: Sci Rep. 2026 Mar 25;16:12713. doi: 10.1038/s41598-026-45170-4 (PMC13090382; doi:10.1038/s41598-026-45170-4)
Supplement: Supplementary file 1 — Supplementary Material 1 [file 41598_2026_45170_MOESM1_ESM.docx]

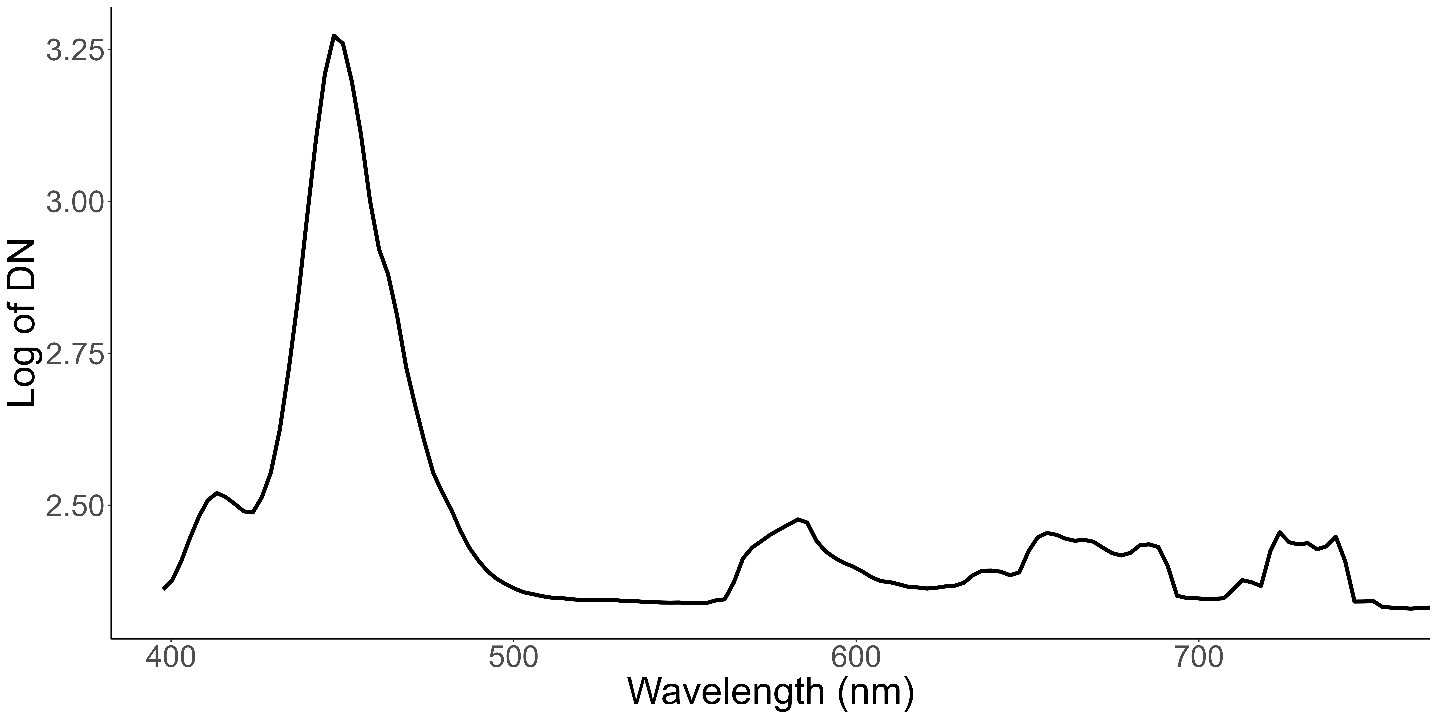


**Supplementary Figure S1.** Raw logarithm transformed spectral signal recorded from the manufacturer-supplied white reference panel provided with the hyperspectral camera system (PTFE calibration target), measured under royal blue excitation (~445 nm). The dominant peak corresponds to the excitation light source. Minor undulations at longer wavelengths reflect wavelength-dependent instrument throughput and detector response at low signal levels rather than additional illumination. Importantly, signal intensity beyond ~750 nm falls to the sensor noise floor, confirming the absence of broadband or infrared emission from the illumination system. These characteristics indicate that red-range features observed in sample spectra arise from wavelength-shifted emission rather than contamination by reflected excitation or extraneous light sources. Accordingly, hyperspectral analyses were restricted to the 418–750 nm range where signal-to-noise ratios were sufficient for reliable interpretation.

**
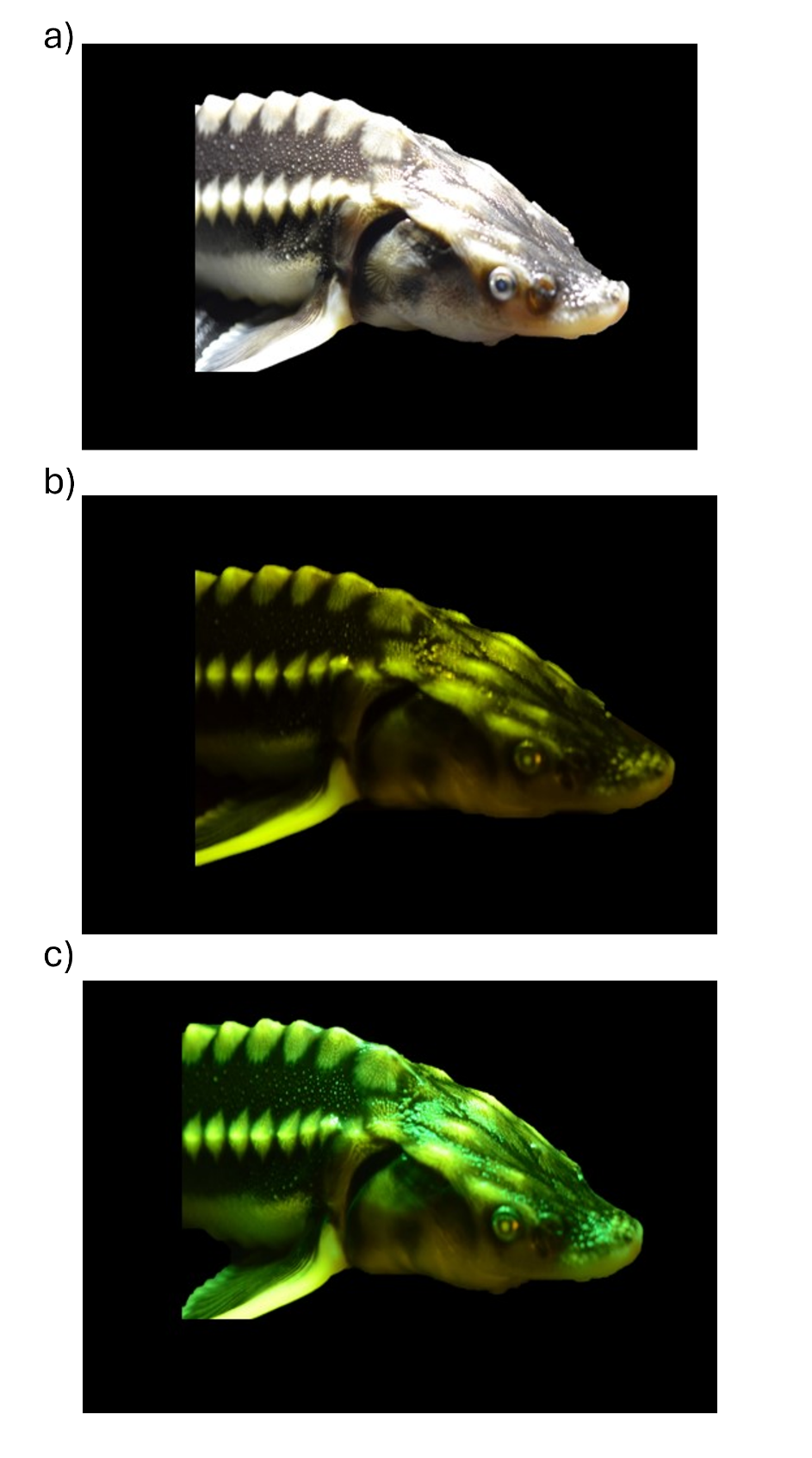
**

**Supplementary Figure S2. Russian sturgeon (Acipenser gueldenstaedtii) – RGB images.** Representative RGB images of Russian sturgeon (*Acipenser gueldenstaedtii*) photographed under different illumination conditions. (a) Ambient white light. (b) Short-wavelength excitation using ultraviolet light (~395 nm) with a long-pass yellow barrier filter mounted on the camera lens. (c) Short-wavelength excitation using royal blue light (~445 nm) with a long-pass yellow barrier filter. Images are shown to illustrate spatial patterns of wavelength-shifted emission under excitation light. RGB images are provided for qualitative visualizaton only; all quantitative spectral analyses were performed using hyperspecral imaging.

**
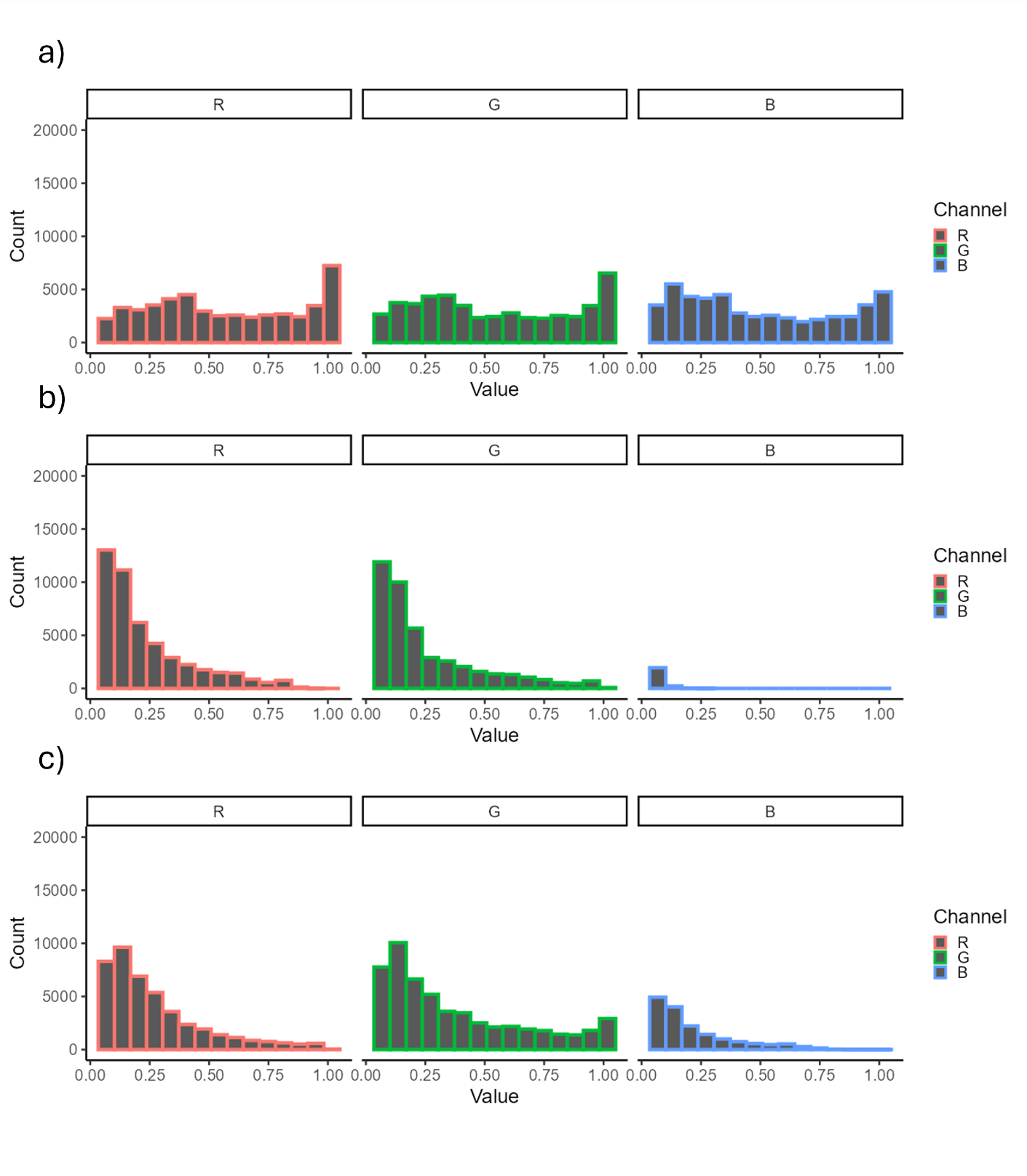
**

**Supplementary Figure S3. Russian sturgeon: RGB pixel-intensity histograms.** Pixel-intensity distributions of the red (R), green (G), and blue (B) channels extracted from representative RGB images of Russian sturgeon (*Acipenser gueldenstaedtii*) shown in Supplementary Figure S2. a) Ambient white light illumination. (b) Ultraviolet excitation (~395 nm) with a yellow long-pass filter. (c) Royal blue excitation (~445 nm) with a yellow long-pass filter. Histograms represent empirical pixel-level intensity distributions from a single representative image and are included to illustrate differences in colour-channel responses under different illumination conditions. They are not intended for statistical comparison or quantitative inference.

**
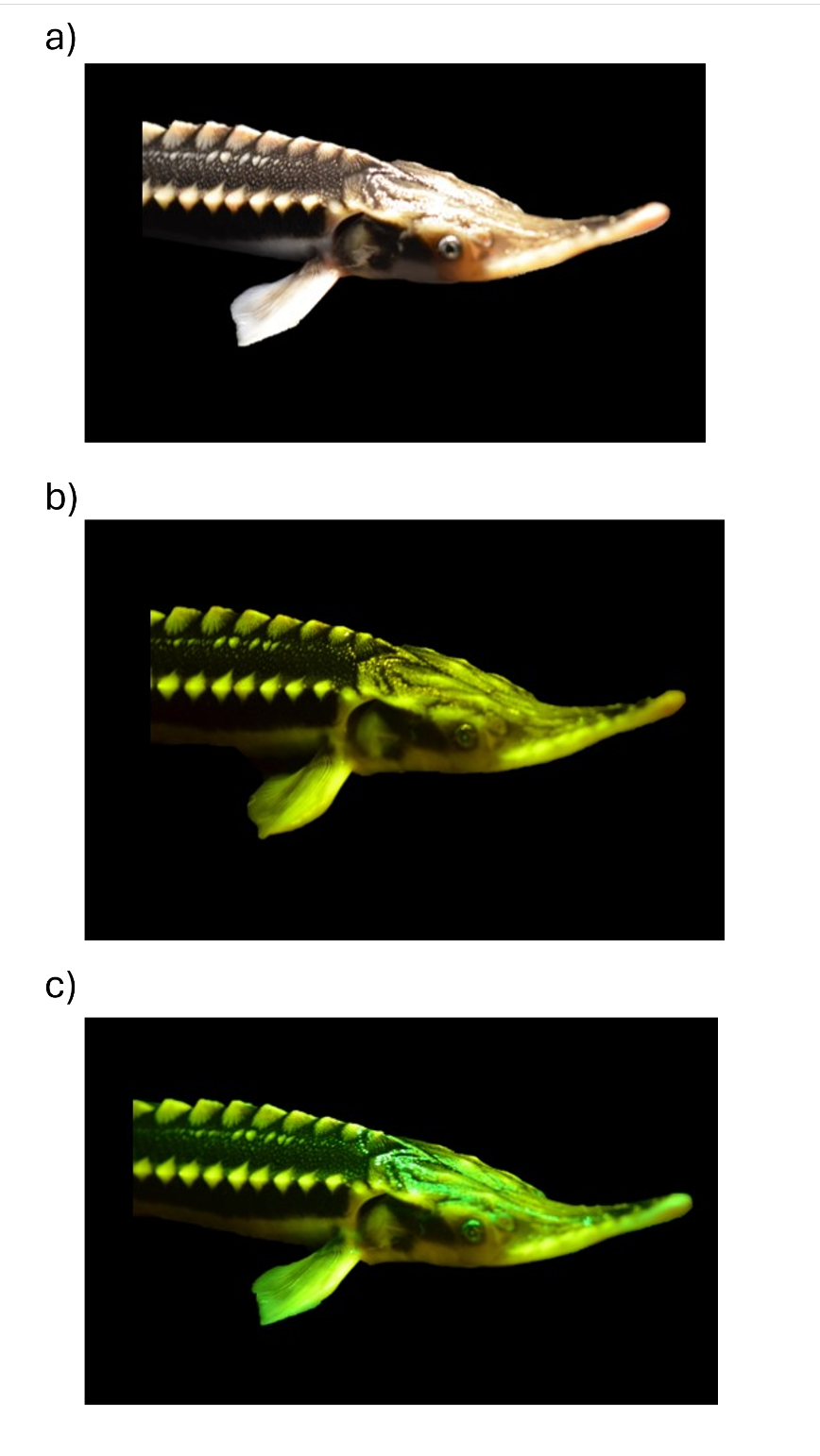
**

**Supplementary Figure S4. Stellate sturgeon RGB images.** Representative RGB images of stellate sturgeon (*Acipenser stellatus*) photographed under different illumination conditions. (a) Ambient white light. (b) Short-wavelength excitation using ultraviolet light (~395 nm) with a long-pass yellow barrier filter mounted on the camera lens. (c) Short-wavelength excitation using royal blue light (~445 nm) with a long-pass yellow barrier filter. Images are shown to illustrate spatial patterns of wavelength-shifted emission under excitation light. RGB images are provided for qualitative visualization only; all quantitative spectral analyses were performed using hyperspectral imaging.

**
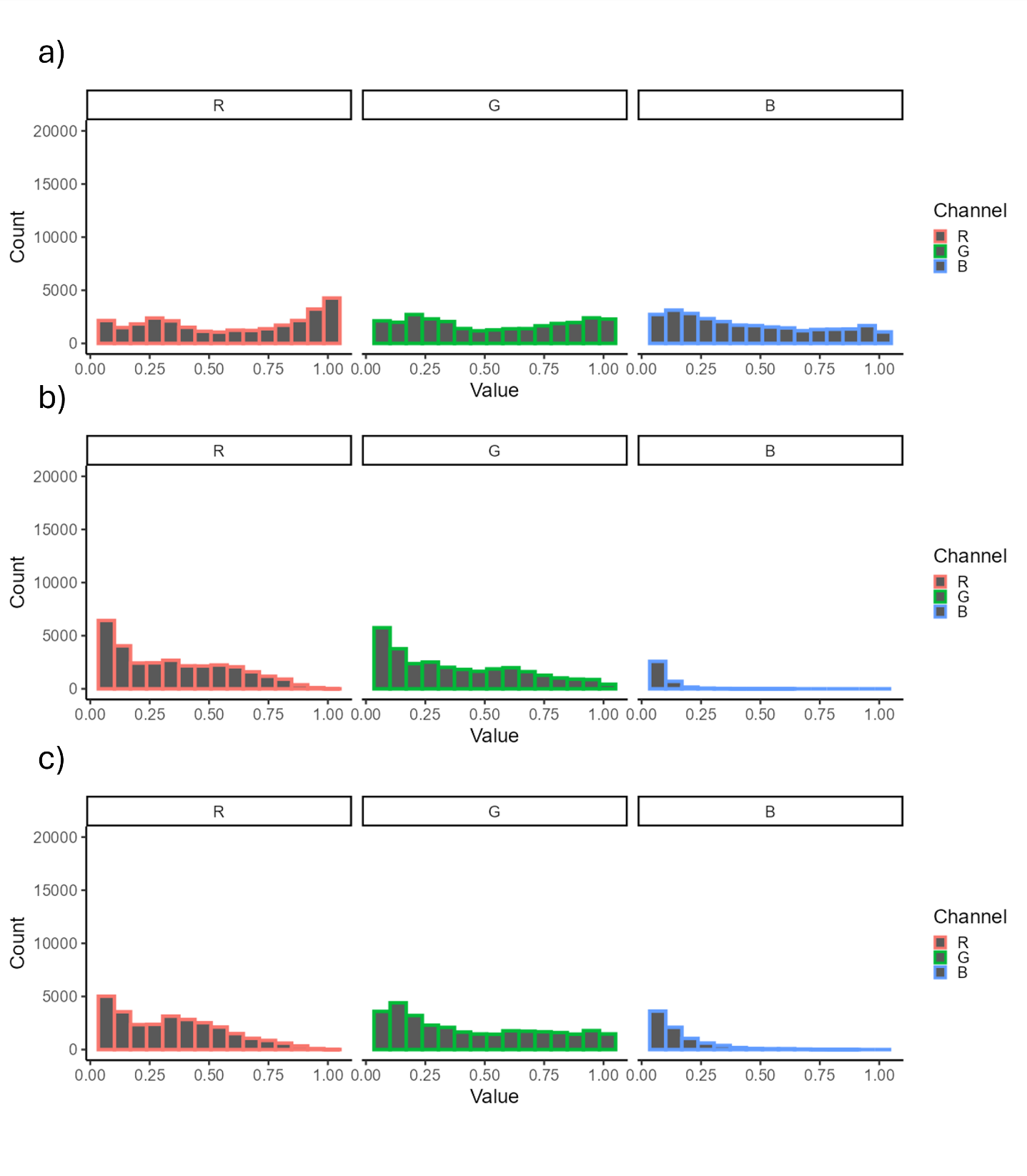
**

**Supplementary Figure S5. Stellate sturgeon: RGB pixel-intensity histograms.** RGB images of stellate sturgeon (*Acipenser stellatus*) shown in Supplementary Figure S4. (a) Ambient white light illumination. (b) Ultraviolet excitation (~395 nm) with a yellow long-pass filter.
(c) Royal blue excitation (~445 nm) with a yellow long-pass filter. Histograms represent empirical pixel-level intensity distributions from a single representative image and are included as an illustrative example of colour-channel responses under different illumination conditions. No statistical comparisons were performed on these data.
